# Pathogenetic and Prognostic Significance of Inactivation of RASSF Proteins in Human Hepatocellular Carcinoma

**DOI:** 10.1155/2012/849874

**Published:** 2012-04-02

**Authors:** Diego F. Calvisi, Matthias Evert, Frank Dombrowski

**Affiliations:** Institut für Pathologie, Universitätsmedizin Greifswald, 17489 Greifswald, Germany

## Abstract

Hepatocellular carcinoma (HCC) is one of the most frequent solid tumors worldwide, with limited treatment options and a dismal prognosis. Thus, there is a strong need to expand the basic and translational research on this deadly disease in order to improve the prognosis of HCC patients. Although the etiologic factors responsible for HCC development have been identified, the molecular pathogenesis of liver cancer remains poorly understood. Recent evidence has shown the frequent downregulation of Ras association domain family (RASSF) proteins both in the early and late stages of hepatocarcinogenesis. Here, we summarize the data available on the pathogenetic role of inactivation of RASSF proteins in liver cancer, the molecular mechanisms responsible for suppression of RASSF proteins in HCC, and the possible clinical implications arising from these discoveries. Altogether, the data indicate that inactivation of the RASSF1A tumor suppressor is ubiquitous in human liver cancer, while downregulation of RASSF2 and RASSF5 proteins is limited to specific HCC subsets. Also, the present findings speak in favour of therapeutic strategies aimed at reexpressing *RASSF1A*, *RASSF2*, and *RASSF5* genes and/or inactivating the RASSF cellular inhibitors for the treatment of human liver cancer.

## 1. Introduction

Hepatocellular carcinoma (HCC) is one of the most frequent tumors, with 0.25-1 million of newly diagnosed cases each year worldwide [1–3]. HCC burden is not distributed evenly throughout the world. Indeed, more than 80% of HCC cases occur in sub-Saharan Africa and Eastern Asia, whereas a much lower HCC incidence characterizes North and South America, Northern Europe, and Oceania [1–3]. Nonetheless, HCC frequency is rapidly growing in low-rate areas as well. In the latter geographic areas, such a rise in HCC occurrence is the result of a combination of factors, including an increasing incidence of cirrhosis caused by alcohol intake, hepatitis C virus (HCV) and hepatitis B (HBV) chronic infection, as well as a general improvement in survival among cirrhosis patients, who are then at risk of developing HCC [1–3]. Furthermore, the rapidly growing number of cryptogenic cirrhosis and HCC cases might be due to a severe form of nonalcoholic fatty liver disease, namely, the nonalcoholic steatohepatitis (NASH) [2].

HCC is a rapidly lethal disease, with an average life expectancy of about 6 months from the time of the diagnosis [1–3]. Like most other solid tumors, surgery plays a fundamental role in its treatment. Surgical resection, local ablation therapies, and liver transplantation are regarded as potentially curative treatment modalities for HCC. However, these therapeutic approaches can be applied only to a limited number of HCC patients since the diagnosis is most often done in the late stages of the disease [1–3]. Furthermore, therapies with pharmacological agents or alternative strategies do not substantially improve the prognosis of patients in which HCC is unresectable [1–3]. Targeted therapies are an innovative and emerging tool to selectively kill cancer cells while sparing the normal, unaffected tissue and thus might be useful for the treatment of human HCC. The effectiveness of targeted therapies against HCC has been recently envisaged by the significantly higher survival length of patients treated with the multikinase inhibitor Sorafenib compared with that of patients treated with placebo [4]. However, the increased life expectancy of Sorafenib-treated patients is limited to about three months, implying that Sorafenib alone cannot substantially modify the prognosis of patients with advanced HCC [4]. This emphasizes the need to investigate the contribution of different signaling pathways to tumor development and progression in human HCC in order to identify novel prognostic markers and molecular targets for its early diagnosis, chemoprevention, and treatment.

Although epidemiologic studies have identified the major risk factors, the molecular pathogenesis of HCC remains largely unknown. It is presumed that development and progression of HCC are the consequence of cumulative genetic and epigenetic events, similar to those described in other solid tumors [5]. Among the most frequently involved tumor suppressor genes in HCCs are *pRb*, *p53*, *M6P/IGF2* receptor, and *E-cadherin* [2, 5]. Oncogenic activation of *c-Myc*, *Cyclin D1*, and **β*-catenin* genes has also been detected in various subsets of HCC [2, 5]. Importantly, unrestrained activation of the Ras/mitogen-activated protein kinase (MAPK) pathway seems to play a major role both in liver malignant transformation and tumor progression [6]. Ras proteins are members of a family of small-guanosine-triphosphate- (GTP-) regulated molecular switches for signaling pathways that modulate cell growth, survival, and migration [7, 8]. Once activated, Ras induces the protein kinase activity of RAF kinase. Raf phosphorylates and activates MAPK kinase kinase (MEK), which subsequently phosphorylates and activates extracellular signal-regulated kinase (ERK), ultimately leading to the upregulation of downstream targets involved in cell proliferation, survival, migration, and invasion [7, 8]. Recent studies have shown that Ras/MAPK pathway is upregulated by multiple factors in HCC in the absence of Ras mutations, including downregulation of the Ras GTPase activating proteins, loss of the ERK inhibitor dual specificity phosphatase 1 (DUSP1), and inactivation of members of the Ras association domain family (RASSF) proteins [6, 9, 10]. Thus, it is plausible to believe that downregulation of cellular inhibitors of Ras may be a key and alternative mechanism leading to the propagation of the Ras signaling in a context of wild-type *Ras* in human HCC.

## 2. RASSF Proteins Status in Human HCC

Due to the widely recognized tumor suppressive role of RASSF1A in carcinogenesis [11–15], numerous studies have investigated its levels in human liver cancer and related them to the clinicopathological parameters. RASSF1A was found to be frequently and progressively downregulated in nontumorous surrounding livers, dysplastic nodules, and HCC when compared with normal (disease-free) livers, with the lowest levels being detected in the tumors. In most investigations, reduced levels of RASSF1A were found to be ubiquitous in HCC regardless of the etiologic factors associated with tumor development (hepatitis B or C chronic infection, alcohol consumption, exposure to food contaminated by aflatoxin B1, etc.), strongly suggesting that universal inactivation of RASSF1A in liver cancer is required for hepatocarcinogenesis [6, 16–22]. Of note, it has been shown [23] that inactivation of *RASSF1A* gene by promoter hypermethylation is already a frequent event in liver fibrosis and cirrhosis, conditions that often precede the development of HCC, but not in hepatocellular adenoma (HCA). Thus, the latter study suggests that RASSF1A hypermethylation occurs early during hepatocarcinogenesis and could be useful as a marker to help discriminating between HCA and HCC [23]. Furthermore, a recent report indicates that reduced RASSF1A protein expression is related to TNM stage, alpha-fetoprotein levels, and the presence of metastasis, portal vein emboli, capsular infiltration, and multiple tumor nodes, implying that assessment of RASSF1A levels might be helpful also for prognosis prediction in human HCC [24].

Different from RASSF1A, the other main RASSF1 isoform (RASSF1C) was found to be expressed at similar levels in normal livers, HCC, and corresponding nontumorous surrounding livers [6]. As concerns RASSF2, its downregulation was detected only at tumor stage and was closely associated with elevated serum alpha-fetoprotein level, but not significantly with clinical stage and hepatic fibrosis [25, 26]. Levels of the main isoforms of RASSF5 (also known as novel Ras effector 1 or NORE1) were also investigated in HCC. The analysis of a large panel of HCC samples showed that NORE1A levels were significantly lower in liver tumors characterized by a poorer outcome (as defined by a patients' length of survival shorter than 3 years after partial liver resection) when compared with HCC with a better prognosis (survival longer than 3 years) [6]. Thus, downregulation of NORE1A seems to be involved in liver tumor progression and biologic aggressiveness. Low levels of the other RASSF5 isoform, NORE1B, were also described in about 60% of the investigated HCC [27]. In the latter tumor collection, downregulation of the *NORE1B* gene did not correlate with tumor grade or stage or the etiology of the disease [27]. Based on this body evidence, it is tempting to speculate that NORE1A and NORE1B may possess distinct biologic activities in liver (cancer) cells.

## 3. Regulation of Expression and Activity of RASSF Proteins

RASSF1A levels and activity are presumably regulated by numerous and complex mechanisms in HCC. These mechanisms include epigenetic silencing of the gene and posttranslational modifications of the protein, which affect RASSF1A stability and half-life. In accordance with a pioneering study on lung cancer and several cancer-derived cell lines, including the HepG2 hepatoma cells [28], epigenetic *RASSF1A* suppression by hypermethylation of its promoter has been suggested as the main mechanism contributing to RASSF1A downregulation in several cancer types, including HCC [6, 11–24]. In the latter disease, promoter hypermethylation of *RASSF1A* gene was shown to occur both in nonneoplastic surrounding livers and HCC, with higher degree of methylation being detected at tumor stage [6, 16–24]. In particular, an intriguing study showed that, in the hepatitic liver (affected by chronic hepatitis and/or cirrhosis, dysplastic nodules, or HCC), the *RASSF1A* gene promoter was extensively methylated, with a methylation degree that increased from regenerative conditions (cirrhosis) to dysplastic nodules, to HCC [21]. Of note, the level of methylation at *RASSF1A* promoter gradually increased by ageing in the nondiseased liver as well [21]. These data suggest the existence of an age-related phenomenon leading to the development and expansion of an epigenetically methylated hepatocyte subpopulation, which might be connected to hepatocarcinogenesis. In another investigation, the *RASSF1A* gene exhibited a weak but clearly detectable methylation signal in normal liver tissue and focal nodular hyperplasia specimens in 57% and 70% of cases, respectively, using sensitive qualitative assay conditions [20]. By using a stringent threshold, none of the normal tissue or focal nodular hyperplasia specimens was methylation-positive, whereas 85% of the hepatocellular carcinoma biopsies were still positive for *RASSF1A* gene hypermethylation [20]. Furthermore, *RASSF1A* methylation degree possessed the highest discriminatory power between HCC and nonmalignant livers [20]. Although the number of investigated specimens was limited, this investigation strongly suggests the use of quantitative real-time PCR-based assays for the assessment of *RASSF1A* promoter methylation status, which might be highly helpful for the discrimination between frankly malignant and nonmalignant liver lesions. In a large-scale study conducted in China, it was found that *RASSF1A *promoter hypermethylation precedes the occurrence of other epigenetic and genetic alterations, such as hypermethylation of *p16INK4A* promoter and mutations of the *p53* gene [29]. Based on these data, the authors suggest that RASSF1A could represent a potential target in preventing malignant transformation of hepatocytes [29]. In addition, promoter methylation of *RASSF1A* gene has been frequently detected in livers affected by hereditary haemochromatosis, a predisposing condition for the development of HCC [30]. Altogether, these data substantiate the role of RASSF1A inactivation both in early and late steps of liver carcinogenesis. Of note, promoter hypermethylation at the *RASSF1A* gene CpG island was found to be frequently associated with additional epigenetic and genetic alterations. In mammary epithelial cells, it has been shown that *RASSF1A* gene inactivation is associated with deacetylation and lysine 9 trimethylation of histone H3 and an impaired binding of Sp1 at the *RASSF1A* promoter. These epigenetic events precede the occurrence of DNA methylation spreading in the *RASSF1A* promoter [31]. Thus, these data suggest that histone modifications may trigger de novo DNA methylation of the *RASSF1A* promoter in epithelial cells. Similar to that described in mammary epithelial cells, epigenetic silencing of *RASSF1A* gene was demonstrated to depend on promoter hypermethylation and histone H3-K9 methylation in human HCC samples [32]. Furthermore, loss of heterozygosity at the lung cancer tumor suppressor locus 3p21.3, where *RASSF1A* is located, was frequently detected in HCC specimens in which the *RASSF1A* promoter was hypermethylated [6, 33]. The two-hit mode (promoter hypermethylation and loss of heterozygosity) of gene inactivation has been described also for other liver tumor suppressor genes, such as *SOCS1-3* and *DUSP1* [6, 9]. As concerns the relationship between *RASSF1A* promoter hypermethylation and the clinicopathological parameters of HCC patients, it has been shown that *RASSF1A* epigenetic silencing is significantly associated with the levels of DNA adducts generated by aflatoxin B1 (AFB1), a mycotoxin with hepatocarcinogenic potential produced by the fungus Aspergillus Flavus [16]. It is important to underline the fact that AFB1 protumorigenic potential in the liver has been attributed to AFB1 mutagenic properties over the p53 tumor suppressor gene so far [34, 35]. The significant association between AFB1 adducts and *RASSF1A* epigenetic inactivation indicates that AFB1 might initiate hepatocarcinogenesis via additional molecular mechanisms independent of p53, including the suppression of the *RASSF1A* gene.

Besides epigenetic silencing and/or genetic loss, posttranslational mechanisms such as microRNA-driven suppression and ubiquitin-dependent proteolysis are also involved in RASSF1A inactivation in HCC. In particular, microRNA-602 has been demonstrated to negatively regulate RASSF1A levels in the HepG2 liver cancer cell line [36]. Also, levels of microRNA-602 were inversely correlated with those of RASSF1A in normal livers, HCC, and corresponding nontumorous surrounding livers, further supporting a role of microRNA-602 in the downregulation of RASSF1A in human liver cancer [36]. Another way of RASSF1A inactivation has been originally described in various cell lines. In these cells, the S-phase kinase-associated protein 2 (SKP2), an oncogenic subunit of the Skp1-Cul1-F-box ubiquitin ligase complex, interacts with ubiquitinates and promotes the degradation of RASSF1A at the G1-S transition of the cell cycle [37]. The SKP2-dependent destruction of RASSF1A requires phosphorylation of RASSF1A on serine-203 by the cyclin D-cyclin-dependent kinase 4 [37]. In human HCC, it has been found that SKP2-dependent proteosomal degradation occurs mainly in tumors characterized by biological and clinical aggressiveness [38]. Also, SKP2-driven ubiquitination and *RASSF1A* epigenetic silencing represent two mutually exclusive mechanisms responsible for RASSF1A inactivation in human HCC [38]. The data obtained in the human HCC sample collection were further substantiated *in vitro*. Indeed, transfection of SKI human HCC cells (expressing low SKP2 levels) with wild-type *SKP2 *cDNA increased the proliferation rate proportionally to SKP2 expression, concomitantly triggering downregulation of multiple tumor suppressor proteins, including P21^WAF1^, P27^KIP1^, P57^KIP2^, P130, FOXO1, and RASSF1A [38]. The proteosomal degradation of the aforementioned proteins was abolished by the treatment with proteosomal inhibitors [38]. Conversely, siRNA-induced knockdown of *SKP2* led to growth restraint of HuH7 human HCC cells (expressing high SKP2 levels), which was paralleled by increase in the levels of P21^WAF1^, P27^KIP1^, P57^KIP2^, P130, FOXO1, and RASSF1A proteins [38]. Thus, these findings suggest that the SKP2-mediated degradation of RASSF1A plays an important role in cell proliferation and survival. Finally, it has been found that the connector enhancer of KSR 1 (*CNK1*) gene, which interacts with RASSF1A and augments RASSF1A-induced cell death [39], is also often epigenetically silenced in human HCC [25].

Altogether, the present findings indicate that multiple mechanisms might play a role in RASSF1A inactivation in human HCC, further substantiating the need of RASSF1A silencing for liver cancer development and progression.

As concerns RASSF2, RASSF4, and RASSF5 isoforms (NORE1A and NORE1B), promoter hypermethylation seems to be the prominent mechanism responsible for their inactivation in liver cancer [6, 25, 27]. Indeed, epigenetic silencing of *RASSF2*, *RASSF4*, and *RASSF5* genes was inversely associated with low mRNA levels of the same genes [6, 25, 27].

## 4. Role of RASSF Proteins in Liver Cancer

Different from other tumor types, the biologic role of RASSF1A has been only minimally investigated in human liver cancer. Some interesting hints on the functional consequences of RASSF1A and NORE1A inactivation on hepatocarcinogenesis were obtained by analyzing a large collection of human HCC specimens [6]. In the latter samples, HCC displayed significantly lower levels of RASSF1A/H-Ras complexes compared with normal livers, indicating that the ability of RASSF1A to bind H-Ras is impaired in HCC. In contrast, RASSF1A/H-Ras complexes were increased in nontumorous surrounding livers, showing that RASSF1A is efficiently bound to H-Ras, thus presumably inhibiting H-Ras activity, at the preneoplastic stage. Furthermore, RASSF1A/NORE1A complexes were found only in the surrounding livers but not in the normal livers or HCC [6]. Because RASSF1A induces apoptosis through heterodimerization with NORE1A [40], these data indicate that RASSF1A-mediated cell death is abrogated in human HCC. Since the induction of RASSF1A and NORE1A leads to activation of MST1 and MST2 proteins [40], the levels of activated MST1 and MST2 were assessed. MST1 and MST2 proteins were phosphorylated in all surrounding nonneoplastic livers in association with caspase 3 cleavage [6]. Accordingly, protein levels of activated SEK1/MMK4-JNK and p38MAPK were low or absent in HCCs without MST1 and MST2 phosphorylation [6], consistent with the notion that MST1 and MST2 are upstream inducers of JNK and p38 MAPK proapoptotic pathways. Besides inducing apoptosis, MST1 and MST2 are crucial regulators of the Hippo signaling pathway. The latter is a conserved signalling cascade involved in the regulation of organ growth in *Drosophila *and vertebrates. In this cascade, MST1 and MST2 form a kinase cascade that is able to phosphorylate at the Ser127 residue the YAP oncoprotein, involved in unconstrained liver growth, leading to YAP inactivation [41–44]. The importance of the Hippo pathway in preventing hepatocarcinogenesis is underscored by the observations that disruption of the Hippo cascade associated with YAP activation triggers liver cancer development in the mouse. Indeed, liver-specific ablation of mouse *WW45* (homolog of the human *SAV1*) gene, an adaptor for the MST kinases, led to increased liver size and expansion of hepatic oval cells and, eventually, liver cancer development [45]. A similar growth effect and the unconstrained expansion of progenitor cells in the mouse liver resulted either from the combined deletion of MST1 and MST2 kinases [46–48] or overexpression of the YAP oncoprotein [49, 50]. Thus, due to its role as a positive regulator of the MST1 and MST2 kinases [11–15] and as inhibitor of MST1 and MST2 dephosphorylation [51], RASSF1A might play a crucial role in preventing liver malignant transformation.

In a recent investigation, transfection of the wild-type form of *RASSF1A* in the QGY-7703 human HCC cell line (expressing low levels of RASSF1A) resulted in fewer and smaller clones, decreased xenograft tumor volume and weight, and led to G1/S arrest both *in vitro* and *in vivo* when compared with cells transfected with the empty plasmid [52]. At the molecular level, transfection of wild-type *RASSF1A* resulted in decreased protein levels of cyclin D1. In addition, forced overexpression of wild-type *RASSF1A* triggered cell growth inhibition and increase in the percentage of cells in the sub-G1 phase following the treatment with mitomycin [52]. A novel proapoptotic pathway connecting RASSF1A to Bax via the Bax binding protein, modulator of apoptosis-1 (MOAP-1), has been described [53]. In this pathway, RASSF1A and MOAP-1 interact directly, and RASSF1A can activate Bax via MOAP-1, thus inducing cell death [53]. Of note, this pathway is impaired in most human liver cancer specimens, due both to inhibition of RASSF1A [53] and epigenetic silencing of *MOAP-1* (Calvisi et al., unpublished results), indicating that loss of RASSF1A-driven apoptosis might be an important molecular event in hepatocarcinogenesis.

Taken together, these data indicate that RASSF1A might exert its tumor suppressive activity on malignant hepatocytes by both inhibiting proliferation and stimulating apoptosis.

The role(s) of NORE1A and NORE1B have also been studied in human HCC. As concerns NORE1B, a microarray study was performed to identify its putative targets in the HEK-293T renal cell line [53]. A series of transcriptional alterations due to NORE1A induction were observed. Among the genes that showed some of the strongest changes in the microarray assay were eukaryotic translation elongation factor 2 (*EEF2*) and spermidine/spermine N1-acetyltransferase 1 (*SAT1*), whose levels were suppressed following *NORE1A* overexpression, and *p*21^*CIP*1^, which was instead upregulated [54]. Further analysis showed that, in human HCC samples, *NORE1A* gene expression directly correlated with *p*21^*CIP*1^ and inversely correlated with *EEF2* and *SAT1* expression [54]. EEF2 is a translation factor that mediates ribosomal translocation during peptide chain elongation and is activated by mitogenic stimuli [55]. *EEF2* is overexpressed in many tumor types and seems to play an important role in rendering tumor cells resistant to the translation-suppressing effects of hypoxia [56]. SAT1 is instead a spermidine kinase that plays a key function in the regulation of the intracellular levels of polyamines [57]. Polyamines play an important role in neoplastic growth, and polyamine synthesis inhibitors are of interest as chemopreventive agents [58]. Microarray analysis has been also performed to determine the signaling profile of RASSF1A in nonsmall cell lung cancer and neuroblastoma [59]. Noticeably, although RASSF1A sequence is 50% identical to that of NORE1A, the two proteins promoted quite different alterations in gene expression [54, 59]. Indeed, SAT1 was the only target identified by both RASSF1A and NORE1A, thus confirming the hypothesis that the functions of NORE1A and RASSF1A are likely to be quite distinct. Several other upregulated targets following NORE1A overexpression that were identified in the array have also been associated with promotion of cell death and induction of growth suppression [54]. Among them, *BTG*3 is a putative tumor suppressor gene and a target of p53 [60], whereas *PDCD2* has been implicated in apoptosis and proliferation control [61]. Thus, NORE1A promotes a number of alterations in transcription that might be involved in the repression of transformation. Nevertheless, the most interesting effect of NORE1A that was identified in the study was the upregulation of the *p*21^*CIP*1^ tumor suppressor gene [54]. The induction of *p*21^*CIP*1^ by NORE1A might explain the ability of NORE1A to induce G1 cell cycle arrest [62], since *p*21^CIP1^ has been shown to block the cell cycle at G1 by inhibiting cdk2 [63]. As mentioned above, the examination of a panel of human HCC showed that loss of NORE1A expression correlated closely with downregulation of *p*21^*CIP*1^ expression [54]. These findings further substantiate the existence of a physiologic link between NORE1A and *p*21^CIP1^ in liver cancer. Moreover, it was demonstrated that NORE1A could only activate *p*21^CIP1^ in a wild-type *p53*-harboring tumor cell line, suggesting the requirement of a nonmutated *p53* gene for the transcriptional induction of *p*21^CIP1^  mediated by NORE1A [54]. Thus, the data indicate that NORE1A is involved in the modulation of one of the major human tumor suppressor pathways. This conclusion was further supported by the observation that mutations of *p53* gene and the inactivation of NORE1A were mutually exclusive events in human HCC [54]. The molecular mechanism(s) by which NORE1A can modulate p53 activity remains unknown. However, an increase of p53 in the nuclear compartment accompanied transfection of *NORE1A* in HuH6 human HCC cells in the same investigation. Thus, it is tempting to hypothesize that NORE1A promotes the nuclear localization of p53 via some posttranslational modification, such as phosphorylation or acetylation.

The role of NORE1B in liver cancer was also investigated [64]. In hepatocyte and hepatoma cell lines, *NORE1B*, *NORE1A*, and *RASSF1A* overexpression led to increase the percentage of cells in G0-G1 at the expense of the S-phase fraction [64]. Furthermore, *NORE1B* and *RASSF1A* insertion in hepatocyte lines resulted in an additional increase in the G2-M fraction, with consequent delay of cell cycle progression and suppression of cell growth. The molecular mechanisms whereby NORE1B reduces the cells in S-phase fraction have not been identified, although the SARAH domain and, to some extent, the RA domain of NORE1B were shown to be essential for growth suppression [64]. Another important discovery was that NORE1B antagonized c-Myc/Ha-Ras-induced transformation of embryonal cells [64]. Of note, *RASSF1A* alone was unable to antagonize cell transformation but enhanced greatly the *NORE1B* effect, which indicates cooperation of these genes. In accordance with the latter finding, the authors found that the NORE1B protein interacts closely with RASSF1A, as determined with fluorescence resonance energy transfer [64]. In further experiments, cell cycle delay by *NORE1B* overexpression was equally effective in hepatocyte cell lines with wild-type or mutant *Ras*, suggesting that NORE1B does not interact with Ras in order to exert its tumor suppressive function [64].

## 5. RASSF Proteins in Experimental Hepatocarcinogenesis

Few studies have investigated the status of RASSF proteins in experimental models of hepatocarcinogenesis to date.

The DNA methylation patterns of *Rassf1a* gene were investigated in the early phase of rat hepatocarcinogenesis induced by a choline-deficient L-amino acid-defined (CDAA) diet [65]. The livers of rats fed the CDAA diet for 4 and 8 days and 3 weeks were methylated in the *Rassf1a* gene promoter, while normal livers were all unmethylated. These results indicate that gene-specific DNA methylation patterns were found in livers of rats after short-term feeding of the CDAA diet, suggesting that gene-specific hypermethylation might be involved in the early phase of rat hepatocarcinogenesis induced by the CDAA diet [65].

The role of cell-cycle-regulating proteins, including Rassf1a, has been evaluated in preneoplastic lesions, dysplastic nodules, and HCC, chemically induced in genetically susceptible Fisher 344 (F344) and resistant Brown Norway (BN) rats [66]. Rassf1a protein levels exhibited no change or low increase in the lesions of F344 rats and consistent rise in dysplastic nodules and HCC of BN rats. Increase in Cks1-SKP2 ligase and proteosomal degradation of cell cycle regulators, including Rassf1a, occurred in F344 but not in BN rat lesions, indicating that posttranslational modifications of cell cycle regulators are under genetic control and contribute to determine a phenotype susceptible to HCC [66]. Furthermore, a gradual increase of Rassf1a/Nore1a/Mst1-driven apoptosis was detected in both rat strains, with highest levels in BN HCC, resulting in significantly higher apoptosis in BN than F344 HCC [67]. Taken together, these data indicate a control of the proapoptotic Rassf1a/Nore1A pathway by HCC susceptibility genes.

In another study, the underlying molecular events associated with tumor-promoting activity of 2-acetylaminofluorene (2-AAF), a complete genotoxic rat hepatocarcinogen, were investigated [68]. The results demonstrate that epigenetic alterations were responsible for driving hepatocarcinogenesis in this model. In particular, preneoplastic and neoplastic liver lesions exhibited increased histone H3 lysine 9 and histone H3 lysine 27 trimethylation in the promoter regions of *Rassf1a*, *p16INK4a*, *Socs1*, *Cdh1*, and *Cx26* tumor suppressor genes, early *Rassf1a* and *p16INK4a* promoter CpG island hypermethylation, and altered microRNA expression in preneoplastic livers of rats exposed to 2-AAF [68]. These changes were accompanied by dysregulation of the balance between cell proliferation and apoptosis, a fundamental protumorigenic event in hepatocarcinogenesis [67].

Altogether, these studies showed the frequent inactivation of Rassf1a either alone or in combination with Nore1a in rat models of hepatocarcinogenesis, implying a universal role of inactivation at least some of the RASSF proteins in liver malignant transformation and tumor progression. Based on these data, it appeared therefore surprising that *Rassf1a* null mice were tumor-prone and spontaneously developed a variety of cancer types, but no HCC or other liver tumors [69]. The lack of HCC development in *Rassf1a* knockout mice was unexpected and remains unclear. Presumably, hepatocarcinogenesis is not triggered by *Rassf1a* inactivation alone, but additional cellular (growth stimuli such as liver regeneration) and molecular (oncogene overexpression, loss of additional tumor suppressor genes) events are required for HCC development in *Rassf1a* knockout mice. A similar situation has been described, for instance, in *Sprouty 2* (a cellular inhibitor of the MAPK pathway) knockout mice. Indeed, inactivation of *Sprouty 2* by overexpression of its dominant negative form in the liver via hydrodynamic transfection was unable to induce significant changes in hepatocytes, whereas the coexpression of the *c-Met* protooncogene resulted in accelerated hepatocarcinogenesis in *Sprouty 2* deficient mice [70]. Therefore, it is plausible that additional cellular and/or molecular stimuli might be necessary for HCC development in *Rassf1a* knockout mice.

## 6. Concluding Remarks

A downregulation of RASSF1A, RASSF2, NORE1A, and NORE1B proteins has been described in human liver cancer. In particular, RASSF1A inactivation is a ubiquitous event and seems to be required for early and late steps of hepatocarcinogenesis, whereas silencing of NORE1A is associated with tumor aggressiveness. Some of the molecular mechanisms whereby RASSF1A, NORE1A, and NORE1B exert their tumor suppressive function have been determined, but presumably these proteins play many other roles in the control of hepatocytes proliferation and survival. In this regard, the study of the crosstalk between the RASSF proteins and the Hippo pathway will presumably provide important insights on liver cancer pathogenesis. A role in hepatocarcinogenesis might be also played by the newly discovered members of the RASSF family, known as N-Terminal RASSF proteins (RASSF7-RASSF10) [71], whose investigation has just begun. The use of appropriate genetically modified models will be highly helpful for the identification and dissection of the RASSF-mediated mechanisms as well as to test therapeutic approaches aimed at reactivating RASSF proteins and/or inactivating RASSF inhibitors, such as the SKP2 ubiquitin ligase, for the treatment of human liver cancer.
